# Improved Work Function of Poly(3,4-ethylenedioxythiophene): Poly(styrenesulfonic acid) and its Effect on Hybrid Silicon/Organic Heterojunction Solar Cells

**DOI:** 10.1186/s11671-016-1759-0

**Published:** 2016-11-30

**Authors:** Xiaojuan Shen, Ling Chen, Jianmei Pan, Yue Hu, Songjun Li, Jie Zhao

**Affiliations:** 1 Institute of Polymer Materials, School of Materials Science & Engineering, Jiangsu University, Zhenjiang, Jiangsu Province 212013 People’s Republic of China; 2 College of Physics, Optoelectronics and Energy & Collaborative Innovation Center of Suzhou Nano Science and Technology, Soochow University, Suzhou, 215006 People’s Republic of China

**Keywords:** Work function, Crystalline silicon, PEDOT:PSS, Carrier recombination, Heterojunction solar cells

## Abstract

Hybrid silicon/organic solar cells have been recently extensively investigated due to their simple structure and low-cost fabrication process. However, the efficiency of the solar cells is greatly limited by the barrier height as well as the carrier recombination at the silicon/organic interface. In this work, hydrochloroplatinic acid (H_2_PtCl_6_) is employed into the poly(3,4-ethlenedioxythiophene):poly(styrenesulfonate) (PEDOT:PSS) solution, and the work function (WF) of the PEDOT:PSS layer has been successfully improved. Based on the Pt-modified PEDOT:PSS layer, the efficiency of the silicon/PEDOT:PSS cell can be increased to 11.46%, corresponding to ~20% enhancement to the one without platinum (Pt) modification. Theoretical and experimental results show that, when increasing the WF of the PEDO:PSS layer, the barrier height between the silicon/PEDOT:PSS interface can be effectively enhanced. Meanwhile, the carrier recombination at the interface is significantly reduced. These results can contribute to better understanding of the interfacial mechanism of silicon/PEDOT:PSS interface, and further improving the device performance of silicon/organic solar cells.

**Electronic supplementary material:**

The online version of this article (doi:10.1186/s11671-016-1759-0) contains supplementary material, which is available to authorized users.

## Background

Crystalline silicon solar cell has enjoyed years of success in the photovoltaic industry due to its excellent properties such as high power conversion efficiency (PCE) and long service life [1–4]. However, the fabrication of the crystalline silicon solar cell is quite complicated and high cost, since the conventional silicon p-n/p-i-n junction has to be formed at a high temperature process [5, 6]. To address the issue, hybrid silicon/organic solar cells, which combine good compatibility with the established silicon technology and the simple fabrication of organics, have been extensively investigated [7–11]. In particular, owing to the high transparence as well as the high conductivity of poly(3,4-ethylenedioxythiophene):poly(styrenesulfonate) (PEDOT:PSS), the hybrid silicon/PEDOT:PSS solar cell is a quite promising candidate for high-efficiency, low-cost photovoltaic application [12]. Over the past years, various approaches have been employed to enhance the performance of this kind of devices, such as silicon surface passivation [13–17], surface morphology controlling [13, 18–21], and rear contact modification [22–25]. Considerable progress has been achieved for the silicon/PEDOT:PSS solar cell, and PCEs of over 11% have been reported by many groups [13, 23, 26–28]. Nevertheless, according to the simulation results, an ultimate PCE over 20% is theoretically possible for the silicon/PEDOT:PSS solar cells, and there are still much room for the performance improvement [29].

The silicon/PEDOT:PSS solar cell is generally assumed to be a Schottky junction solar cell, where the PEDOT:PSS serves as the metallic contact. In the cell, light is predominately absorbed by the silicon, and the generated electron-hole pairs are separated and swept into the proper directions by the driving force of the built-in potential (*V*_bi_) between the silicon/metal interfaces. It has been reported that increasing the *V*_bi_ can successfully improve the silicon/PEDOT:PSS cell performance, such as inserting a high work function (WF) of hole transport layer of MoO_3_ or WO_3_ between the PEDOT:PSS layer and the top grid electrode [27, 30] and doping/undoping the rear contact [23, 25]. Fortunately, unlike the silicon/metal Schottky solar cells, where the barrier height of the silicon/metal is relatively insensitive to the WF of the metal owing to the formation of metal silicides during vacuum metal deposition on silicon [31], tuning the WF of PEDOT:PSS layer might be a direct and efficient approach to increase barrier height. By doping the PEDOT:PSS layer with perfluorinated ionomer (PFI), the WF of the layer has been increased about 0.3 eV, improving the PCE of the silicon/PEDOT:PSS cell form 8.2 to 9.9% [32].

Platinum (Pt) particles have attracted great attention due to its various potential applications, such as a catalyst in fuel cells and solar cells [33, 34] and charge storage medium in memory devices [35]. Pt particles can be easily obtained by thermally decomposed of hexachloroplatinic (IV) acid (H_2_PtCl_6_) [8], which is one of the most readily available soluble compounds of Pt. In this work, we introduce H_2_PtCl_6_ to PEDOT:PSS solution and investigate its effect on the conductivity as well as WF of the fabricated PEDOT:PSS layer. Additionally, the effect on the device performance of corresponding silicon/organic solar cells is also explored. The results contribute to better understanding of the interfacial mechanism of silicon/PEDOT:PSS interface and further improving of the device performance of silicon/organic solar cells.

## Methods

### Materials

PEDOT:PSS solution (CLEVIOS PH 1000), Triton, and hydrochloroplatinic acid were purchased from Aldrich. Hydrofluoric acid (HF) and dimethyl sulfoxide (DMSO) were purchased from Sinopharm Chemical Reagent Co., Ltd., China and used as received. Deionized water (DI) with a resistance of 18 MΩ cm^−1^ was purified using a Nanopure Diamond system.

### PEDOT:PSS Layer Fabrication

PEDOT:PSS solution (CLEVIOS PH 1000) was mixed with Triton (1 wt%) and DMSO (5 wt%). Additionally, in order to increase the WF of PEDOT:PSS layer, various volumes of hydrochloroplatinic acid (H_2_PtCl_6_), which was ~1 mg/ml in water, was also added. The volume ration of H_2_PtCl_6_ was 0, 5, 10 15, and 20%, respectively. The resulting solution was kept stirring for about 2 h and then spin-coated on the substrates at 2500 rpm for 1 min. After that, the layers were sintered at 180 °C for 15 min in a nitrogen atmosphere.

### Device Fabrication

The clean n-type Si (100) substrates with resistivity of 1~3 Ω cm were methylated through a two-step chlorination/alkylation method [7, 36]. After that, PEDOT:PSS with different volume of H_2_PtCl_6_ were spin-coated on the silicon wafer at 2500 rpm for 1 min and then were heated at 180 °C for 15 min in a nitrogen atmosphere. The silver grid electrodes are thermally evaporated through a shadow mask with a 10 × 8 mm^2^ area, and the rear contact was obtained by vacuum thermal evaporating aluminum.

### Characterization

The film conductivity was obtained using a four-point-probe setup. The Kelvin probe miscrope images were recorded with a commercial AFM system (Veeco instruments MultiMode AFM with NanoScope IIIa controller and extender module) operating in LiftMode. X-ray diffraction (XRD) measurement is carried out using an X-ray powder diffractometer (PANAlytical Empyrean). The morphology of the films was observed using a scanning electron microscope (SEM, FEI/Quanta 200 FEG).

A Newport 91160 solar simulator equipped with a 300 W xenon lamp and an air mass (AM) 1.5 G filter was used to generate simulated solar spectrum irradiation source. The irradiation intensity was 100 mW cm^−2^ and calibrated by a Newport standard silicon solar cell 91150. The electrical data were recorded by a Keithley 2612 source meter. The capacitance was characterized with a Wayne Kerr 6500B impedance analyzer.

## Results and Discussion

### Characteristics of PEDOT:PSS Layers

**Fig. 1:**
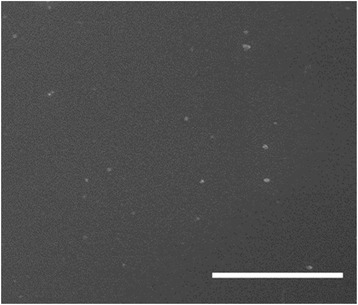
SEM images of PEDOT:PSS layer fabricated with addition of H_2_PtCl_6_. The volume ration of H_2_PtCl_6_ is 10%. (the scale bare 5 μm)

In order to explore the effect of H_2_PtCl_6_, the conductivity as well as the WF of PEDOT:PSS layers fabricated with different volume of H_2_PtCl_6_ in water are investigated. Table 1 summarizes the conductivity as well as surface potential of the fabricated PEDOT:PSS layers. In the PEDOT:PSS solution, the conductive PEDOT grains are surrounded by the insulating PSS shells. After mixing with H_2_PtCl_6_, the Pt compound with a negative charge can be easily adhered onto the PEDOT grains that present a positive charge. Since H_2_PtCl_6_ can be thermally decomposed into Pt particles after sintering, the formed Pt particles are dispersed in PEDOT:PSS layer. Due to the same charge properties (Pt compound vs. PSS shell), the Pt compound can avoid agglomeration upon two aqueous solutions mixing under stirring. As shown in Fig. 1a, the formed Pt particles can be well dispersed in PEDOT:PSS layers without clear aggregation. Additional file 1: Figure S1 shows the XRD spectra of PEDOT:PSS layers fabricated without and with H_2_PtCl_6_. The decreased peaks suggest that the crystallinity of PEDOT:PSS layers with H_2_PtCl_6_ is reduced. Thus, with the H_2_PtCl_6_ addition, the conductivity of the fabricated PEDOT:PSS layer is decreased. When the volume ration is 10%, the conductivity of the layer decreases from 465 to 427 S/cm.

**Table 1:** The conductivity as well as surface potential of PEDOT:PSS layer containing different volumes of H_2_PtCl_6_

Volume (%)	Conductivity (S/cm)	Surface potential (mV)
0	465	160
5	438	15
10	427	−80
15	420	−150
20	409	--

[--] No measurement

The WF of PEDOT:PSS layers are investigated with the SKPM method [34, 35]. The relation between WF of the conductive tip, *Φ*_tip_, and the samples, *Φ*_s_, is given in the following equation.

1 $$ {\varPhi}_{\mathrm{s}} = {\varPhi}_{\mathrm{tip}}-e{V}_{\mathrm{CPD}} $$

**Fig. 2:**
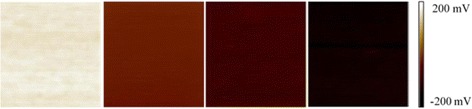
Surface potential images of PEDOT:PSS layers fabricating form PEDOT:PSS solution containing different volumes of H_2_PtCl_6_ probed by SKPM for **a** 0%, **b** 5%, **c** 10%, and **d** 15%. All the measurements are carried out by using the same conductive tip. All the *sale bars* are 1 × 1 μm

Where *e* is the elementary charge and the *V*_CPD_ is the surface potential directly measured by SKPM [37, 38]. The WF variation can be easily observed by comparing the difference of *V*_CPD_. Figure 2 shows the *V*_CPD_ of PEODOT:PSS layers fabricating with different volumes of H_2_PtCl_6_. It can be observed that the *V*_CPD_ becomes more negative along the increasing volume of H_2_PtCl_6_. Here, the decrease of *V*_CPD_ indicates a corresponding increase of WF. When the volume ration is 15%, the *V*_CPD_ of the layer decreases from ~160 mV of the pristine layer to ~-150 mV, which means the WF of the layer increases about 310 mV. There are two possible reasons for the enhanced WF. First, H_2_PtCl_6_ can be thermally decomposed to Pt particles with high WF, and doping into the PEDOT:PSS layer can directly enhance the WF of PEDOT:PSS layer. Second, during the Pt particles formation, the density of PSS shells in the film increased. Additional file 1: Figure S2 shows the absorption spectra of PEDOT:PSS films without and with H_2_PtCl_6_. The absorption peaks at 193 and 225 nm are assignable to π−π* transitions of the PSS benzene ring [39], the increase of peak absorption for PEDOT:PSS layer modified with Pt particles indicates an increase in PSS concentration, which can also increase the WF of the PEDOT:PSS layer [40].

**Fig. 3:**
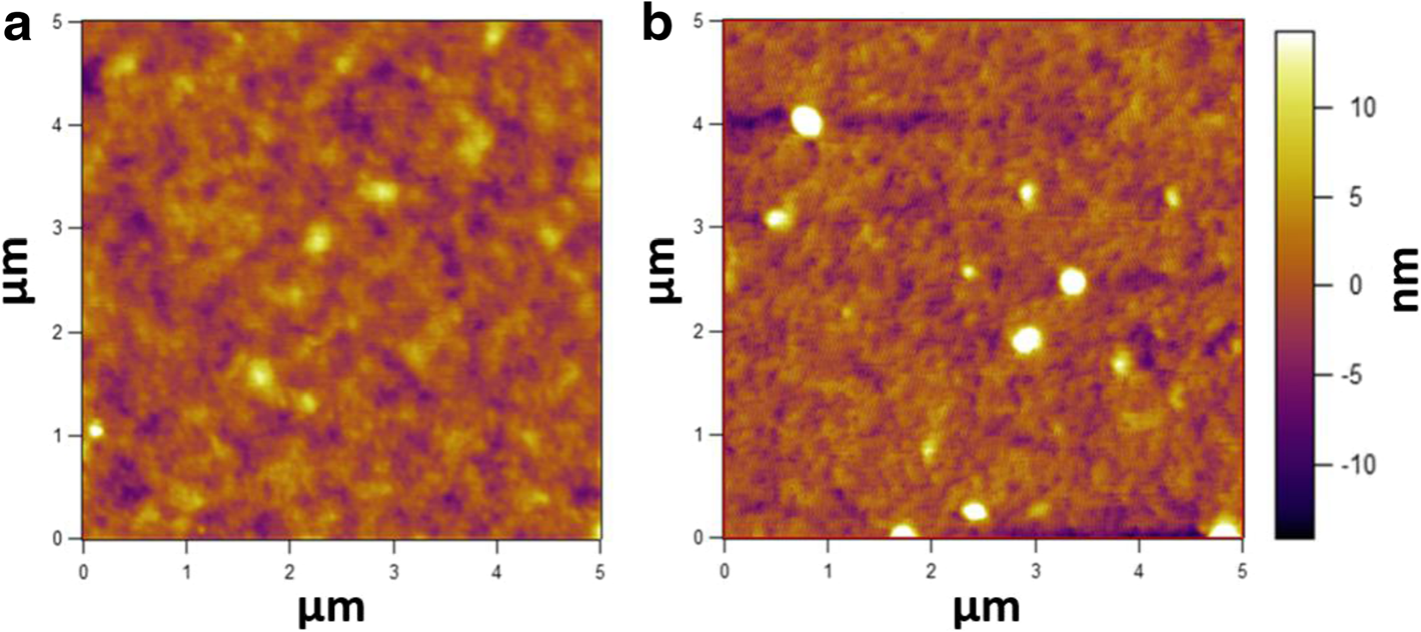
AFM height images of the the PEDOT:PSS layers **a** without H_2_PtCl_6_, RMS = 2.294 nm and **b** with H_2_PtCl_6_, RMS = 3.916 nm. The volume ration of H_2_PtCl_6_ is 10%

In previous report, when PEDOT:PSS was mixed with HAuCl_4_, both of the conductivity and WF of the PEDOT:PSS layer were increased [41]. However, in present study, though the WF of the PEDOT:PSS layer is enhanced, the conductivity of the layer is reduced. The difference may origin from the large formed particles in the layer. As shown in Additional file 1: Figure S3, the diameter of the formed particle is ~200 nm, which could disturb and inhibit 3-dimensional connections between the conducting PEDOT chains [42, 43]. AFM images of PEDOT:PSS layers fabricated with and without H_2_PtCl_6_ have also been measured as shown in Fig. 3. It can be observed that surface of PEDOT:PSS layers with and without Pt particles are similar, and the size of the formed Pt particles in PEDOT:PSS layer is consistent with that of SEM image (Additional file 1: Figure S3).

### Device Performance for Hybrid Silicon/PEDOT:PSS Cells

**Fig. 4:**
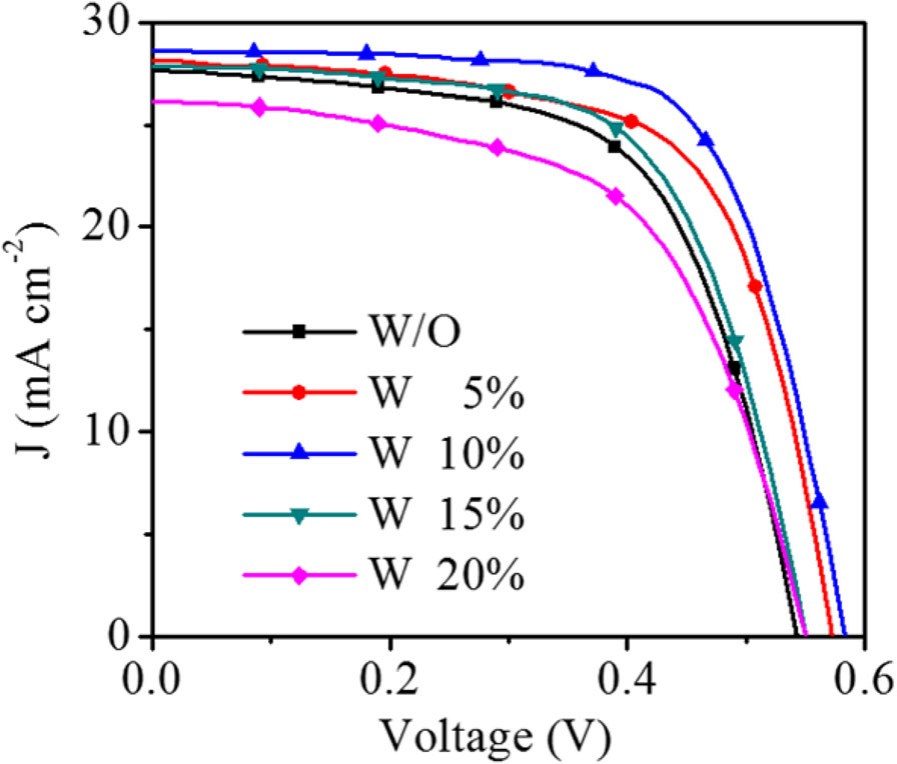
J-V curves of the hybrid silicon/PEDOT:PSS devices fabricating with different volume ration of H_2_PtCl_6_ under AM1.5 simulated illumination at 100 mW cm^-2^

Figure 4 shows the current density (J) vs. voltage (V) characteristics of the hybrid silicon/PEDOT:PSS devices fabricating with different volumes of H_2_PtCl_6_ under AM1.5 simulated illumination at 100 mW cm^-2^. The electric output characteristics of short-circuit current density (*J*_SC_), open-circuit voltage (*V*_OC_), fill factor (FF), and PCE are summarized in Table 2. It could be observed that the performance of devices improve first along the volume ration, peaking at 10% with a PCE of 11.46% which corresponds to ~20% improvement. It should be noted that though the WF of PEDOT:PSS layer is increased along the volume of H_2_PtCl_6_, the conductivity of PEDOT:PSS layer is reduced. At the low volume of H_2_PtCl_6_, this potential drawback can be overwhelmed by the advantage resulting from the enhanced WF, which means employing large *V*_bi_ for charge separation and collection. Thus, the enhancement of the device performance mainly originates from the increase in FF and *V*_OC_, from 0.628 to 0.692 and 0.541 to 0.580 V, respectively. Further increasing the volume, the performance of the device gets degraded. There are two possible reasons for this phenomenon. First, in addition to the decreased conductivity, as shown in Additional file 1: Figure S4, big clusters of particles are formed which could promote carrier recombination between the layer and the metal electrode. Second, the layer roughness which results from the increased Pt particles is also increased. The rougher surface could cause the difficulty of the uniform formation of the Ag electrodes during the thermal evaporation, increasing the contact resistance between the layer and Ag electrode [27]. As a result, when the volume ration of H_2_PtCl_6_ is higher, the performance of the devices is reduced.

**Table 2:** The electric output characteristics of the hybrid silicon/PEDOT:PSS devices fabricating with different volumes of H_2_PtCl_6_

Volume (%)	*J*_sc_ (mA/cm^2^)	*V*_oc_ (V)	FF	PCE (%)
0	0.541	28.08	0.629	9.56
5	0.570	28.39	0.657	10.63
10	0.580	28.57	0.692	11.46
15	0.551	27.85	0.639	9.79
20	0.552	26.14	0.585	8.42

Notably, when the PEDOT:PSS solution is mixed with H_2_PtCl_6_ in water, the layer thickness is decreased. In order to investigate the effect of thickness on the devices, we can compare the devices without or with the water addition. According to the results of the devices with different volumes of H_2_PtCl_6_ (Fig. 4 and Table 2), the optimal volume ration is 10%. Here, adding 10% volume ration of water, the thickness of the PEDOT:PSS layer is decreased from 100 to 89 nm. Nevertheless, as shown in Additional file 1: Figure S5, the J-V characteristics of the devices are similar, the PCE of the device without and with water addition are 9.51% and 9.58%, respectively. No discernible difference in the performance is observed. Hence, we make sure the change is caused by H_2_PtCl_6_.

### Electrical Effect of H_2_PtCl_6_

**Fig. 5:**
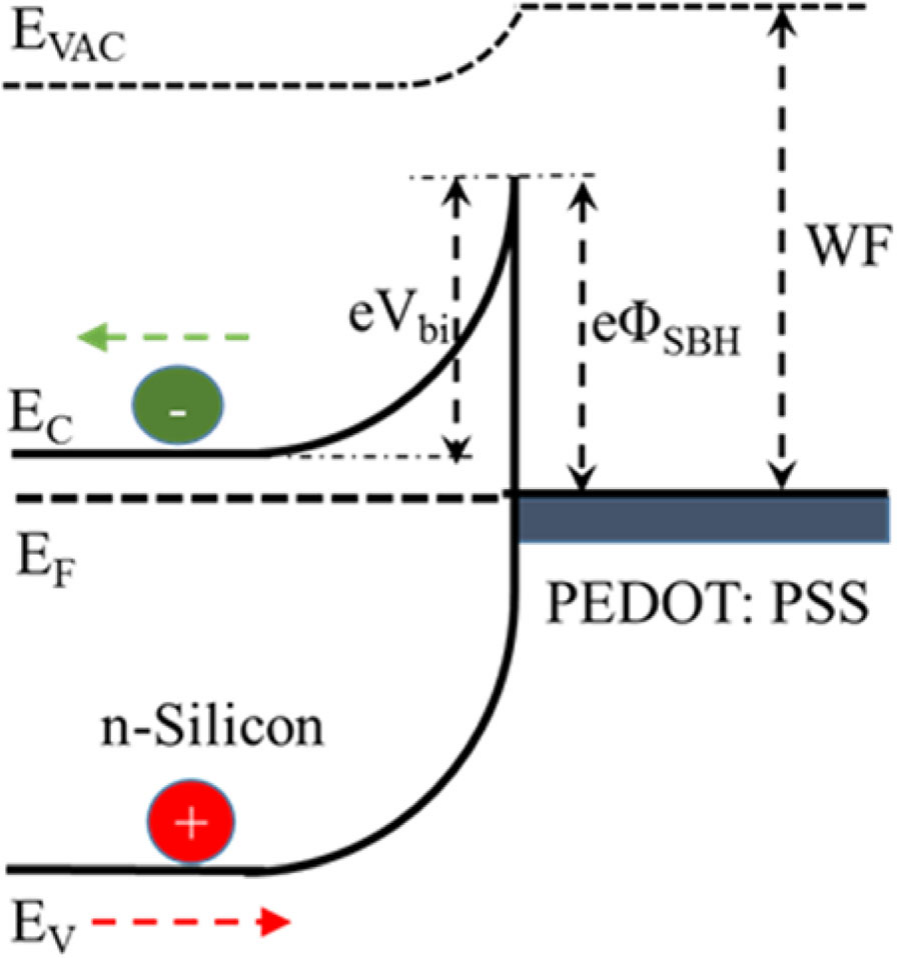
Energy diagram of Schottky solar cell based on n-type silicon/PEDOT:PSS interface. *Φ*_SBH_ is the Schottky barrier height, *E*_F_ is the energy of the Fermi level, *E*_C_ and *E*_V_ are the conduction band and valence band, respectively

**Fig. 6:**
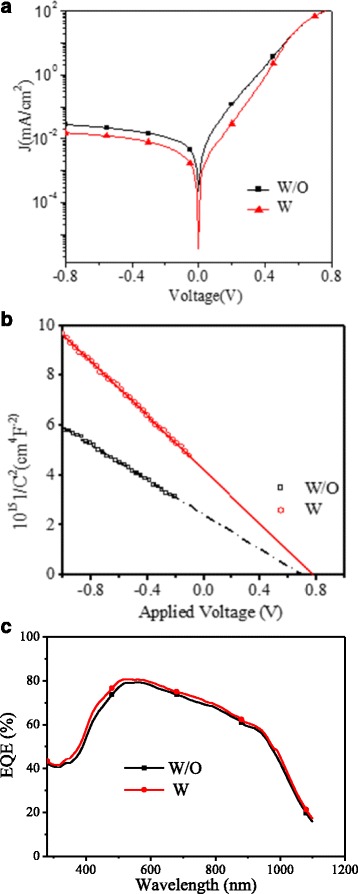
**a** J-V curves of the devices without and with H_2_PtCl_6_ in the dark condition. **b** 1/*C* ^2^–*V* plots of the devices without and with H_2_PtCl_6_. **c** EQE spectra of the devices without and with H_2_PtCl_6_. The volume ration of H_2_PtCl_6_ is 10%

In the hybrid silicon/PEDOT:PSS devices, light is predominately absorbed by the silicon, the generated electron-hole pairs are separated and swept into the proper directions by the driving force of *V*_bi_. So, the *V*_bi_ between the silicon/PEDOT:PSS interface is an important factor which should be high enough to guarantee the charge separation and collection, sweeping charge into the proper directions. Figure 5 illustrates the band energy diagram at the n-type silicon/PEDOT:PSS interface. According to an ideal Schottky-Mott model [44], the Schottky barrier height (*Φ*_SBH_) at the silicon/PEDOT:PSS interface is proportional to the difference between the WF and the electron affinity of silicon (*χ*_Si_) by an equation of *eΦ*_SBH_=WF−*χ*_Si_. Because the *V*_bi_ is related to the *Φ*_SBH_ via the expression of *Φ*_SBH_=*V*_bi_ + e^−1^ kT ln(N_C_/N_D_), where *N*_C_ is the effective density of states in the conduction band and *N*_D_ is the doping level of the semiconductor. In our devices, methylation could successfully passivate the silicon surface, there could be no difference for *N*_C_ and *N*_D_ in the silicon substrates. Therefore, increasing *Φ*_SBH_ means enhancing *V*_bi_. Improving the WF of PEDOT:PSS layer could successfully enlarge the *Φ*_SBH_/*V*_bi_ of the device. After mixing with H_2_PtCl_6_, the WF of PEDOT:PSS layer is improved. In order to confirm the increase of *Φ*_SBH_/*V*_bi_ resulting from the enhanced WF, we analyze the dark J-V data as shown in Fig. 6a with the help of the thermionic emission model [45]:

2 $$ J={J}_{\mathrm{S}}\left( \exp \left(\frac{e}{nkT}V\right)-1\right) $$

3 $$ {J}_{\mathrm{s}}={A}^{*}A{T}^2 exp\left(-\frac{\varPhi_{\mathrm{SBH}}}{kT}\right) $$

Where *J* is the current density, *J*_S_ is the reversed saturation current, *A* is the contact area, *A* ^*^ is the effective Richardson constant (≈252 A cm^−2^ K^−2^ for n-type silicon), *T* is the absolute temperature (298 K), *k* is the Boltzman constant, and *n* is the diode ideality factor. With the H_2_PtCl_6_ addition, the estimated *Φ*_SBH_ of the hybrid silicon/PEDOT:PSS device increases from 0.783 to 0.856 V. In order to further confirm the increase of *Φ*_SBH_/*V*_bi_, the analysis of the C-V measurement is also used, which affords the information about the magnitude of *V*_bi_. According to Anderson’s model [39], the capacitance of the device can be described as 1/*C* ^2^∝(*V*_bi_−*V*) where *C* is capacitance, *V* is the applied voltage, and the value of *V*_bi_ can be extracted from the extrapolation of the linear portion of the 1/*C* ^2^−*V* plots. As shown in Fig. 6b, the *V*_bi_ value for the devices without and with H_2_PtCl_6_ are 0.69 and 0.77 V, respectively, showing 0.11 V increase. Both of the results coming from the dark J-V curves analysis and C-V measurement confirm that increasing the WF of PEDOT:PSS layer can successfully improve the *Φ*_SBH_/*V*_bi_ of the silicon/PEDOT:PSS devices. Since the *V*_OC_ of the silicon/PEDOT:PSS device is directly proportional to the *V*_bi_, the increase in *V*_OC_ of the device with H_2_PtCl_6_ addition can be ascribed to the enhancement of *V*_bi_. In addition, enhancing the *V*_bi_ is also beneficial for the charge separation and collection, largely reducing the recombination loss at the interface, thus the FF of the devices can be increased.

External quantum efficiency (EQE) measures the percentage of incident photons, those eventually result in free charges being collected through the electrodes. Factors beyond light absorption, such as the resistance of electrodes, charge separation, and collection efficiencies, will also affect the magnitude of EQE, which is not sensitive to the wavelength. Here, as shown in Fig. 6c, due to the enhancing charge separation and collection resulting from the improved *V*_bi_, the value of EQE spectrum device with H_2_PtCl_6_ is higher than that of device without H_2_PtCl_6_.

### Minority Carrier Lifetime

**Fig. 7:**
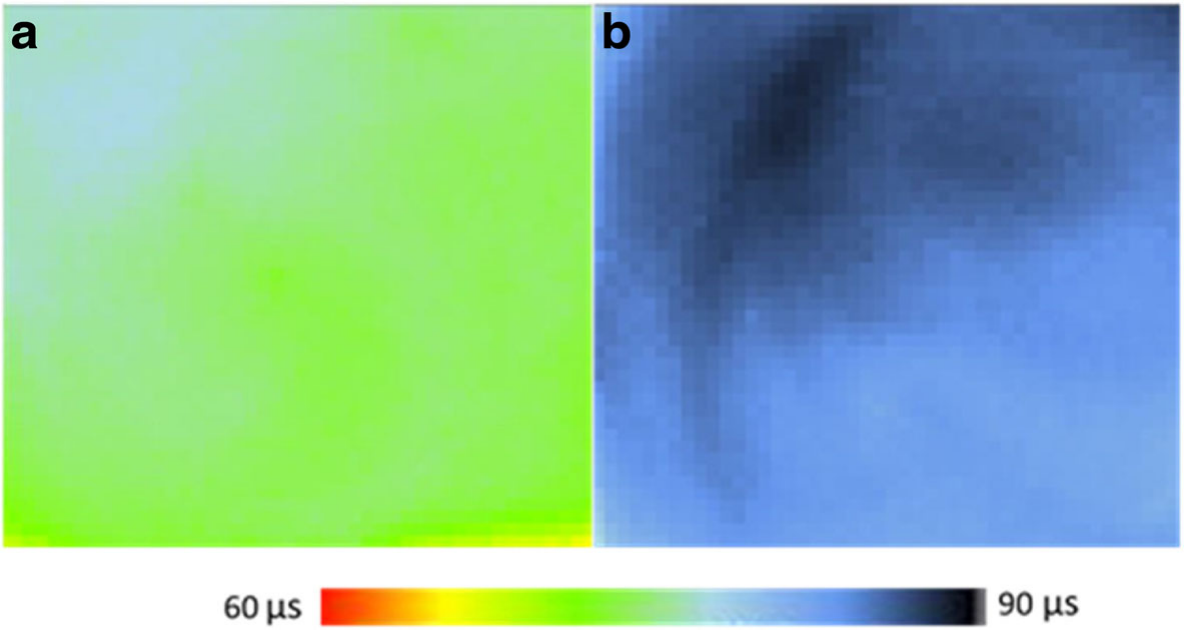
Spatial mapping of minority carrier lifetime measurement for the samples of silicon/PEDOT:PSS **a** without and **b** with H_2_PtCl_6_. The volume ration of H_2_PtCl_6_ is 10%

To further investigate the silicon/PEDOT:PSS interface quality, we conduct a spatial mapping of minority carrier lifetime measurement for the samples. The lifetime mappings of the silicon/PEDOT:PSS samples without and with Pt particles are presented in Fig. 7, which correspond to an average lifetime of 74 and 84 μs, respectively. The increase of the average lifetime suggests that the effective minority carrier lifetime of the device can be improved with H_2_PtCl_6_ addition. Generally, the effective minority carrier lifetime of a silicon solar cell can be expressed as follows [13, 30]:

4 $$ 1/{t}_{\mathrm{eff}}=1/{t}_{\mathrm{bulk}}+2S/W $$

where *t*_eff_ is the effective lifetime, *t*_bulk_ is the bulk recombination lifetime, *S* is the surface recombination rate, and *W* is the wafer thickness. Since *t*_bulk_ is fixed for the same silicon wafer, the increase of measured lifetime (*t*_eff_) reflects a lower surface recombination rate. The carrier recombination rate at the silicon surface is generally proportional to the population of electrons and holes. The introduction of H_2_PtCl_6_ can successively enhance the *Φ*_SBH_/*V*_bi_, which significantly reduces the population of electrons near the silicon surface and transfers them to the electrode; therefore, the carrier recombination at the silicon surface is largely reduced, resulting in the improvement of carrier lifetime. Note that the increase of carrier lifetime is expected to improve the *J*_SC_ of the device. However, the *J*_SC_ of the devices without and with H_2_PtCl_6_ has slight change. As mentioned above, there are two possible reasons for this point. One is that the slight decreased conductivity (465 S/cm without vs. 427 S/cm with H_2_PtCl_6_) of the layer, and the other is the rougher layer which is not beneficial for the charge transfer and collection. The competition between the increased carrier lifetime and the decreased conductivity as well as increased roughness of layer contributes to the slight JSC variation.

## Conclusions

In summary, we have introduced H_2_PtCl_6_ to PEDOT:PSS solution, and the WF of the PEDOT:PSS layer has been successfully improved. The increased WF of Pt-modified PEDOT:PSS layer obviously enhances the *Φ*_SBH_ as well as *V*_bi_ of the silicon/PEDOT:PSS interface, which is beneficial for the charge separation and collection, and greatly suppresses the charge recombination at the silicon/PEDOT:PSS interface. As a result, the PCE of the hybrid silicon/PEDOT:PSS cell increases up to 11.46%, corresponding to ~20% enhancement to the one without Pt modification. Our results contribute to better understanding of the mechanism of silicon/PEDOT:PSS interface and further improving of the device performance of silicon/organic solar cells.

## Additional file

Additional file 1: Supplementary data. (DOC 183 kb)

## Abbreviations

EQE: External quantum efficiency
H_2_PtCl_6_: Hydrochloroplatinic acid
PCE: Power conversion efficiency
PEDOT:PSS: Poly(3,4-ethlenedioxythiophene):poly(styrenesulfonate)
*V*_bi_: Built-in potential
WF: Work function
