# Supplementary material for: Improved Work Function of Poly(3,4-ethylenedioxythiophene): Poly(styrenesulfonic acid) and its Effect on Hybrid Silicon/Organic Heterojunction Solar Cells
Source: Nanoscale Res Lett. 2016 Nov 30;11:532. doi: 10.1186/s11671-016-1759-0 (PMC5131012; doi:10.1186/s11671-016-1759-0)
Supplement: Additional file 1: — Supplementary data. (DOC 183 kb) [file 11671_2016_1759_MOESM1_ESM.doc]

Supplementary data for

Improved work function of poly(3,4-thylenedioxythiophene): poly(styrenesulfonic acid) and its effect on hybrid silcon/organic heterojunction solar cells

Xiaojuan Shen,^a^ Ling Chen,^a^ Jianmei Pan,^a^ Yue Hu,^a^ Songjun Li,*^a^ Jie Zhao*^b^

^a^ Institute of Polymer Materials, School of Materials Science & Engineering, Jiangsu University, Zhenjiang, Jiangsu Province, 212013, P.R. China.

^b^ College of Physics, Optoelectronics and Energy & Collaborative Innovation Center of Suzhou Nano Science and Technology, Soochow University, Suzhou215006, P.R. China.

Xiaojuan Shen. E-mail: xiaojuanshen@ujs.edu.cn

Ling Chen. E-mail: 17788354523@163.com

Jianmei Pan. E-mail: jmpanc@163.com

Yue Hu. E-mail: 812855961@qq.com

Songjun Li. E-mail: lsjchem@ujs.edu.cn

Jie Zhao. E-mail: jzhao@suda.edu.cn


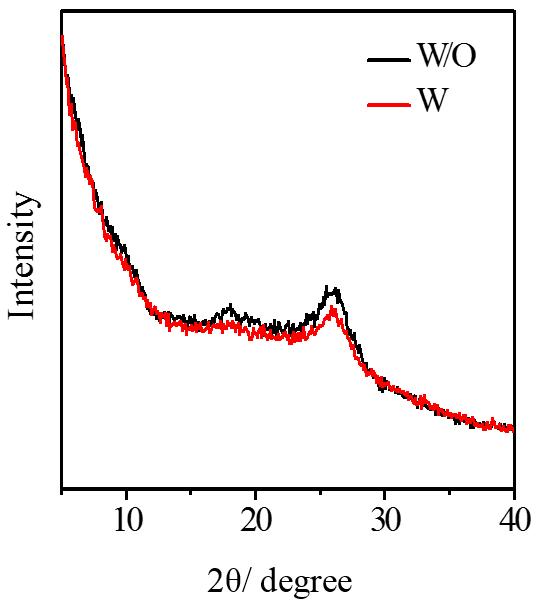


**Fig. S1** XRD spectra of PEDOT:PSS layers without and with H_2_PtCl_6_. The volume ration of H_2_PtCl_6_ is 10%.


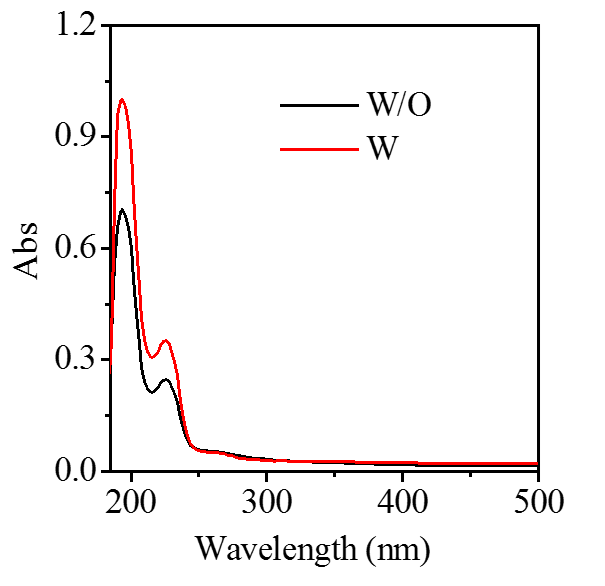


**Fig. S2** Absorption spectrum of PEDOT:PSS layers without and with H_2_PtCl_6_. The volume ration of H_2_PtCl_6_ is 10%.


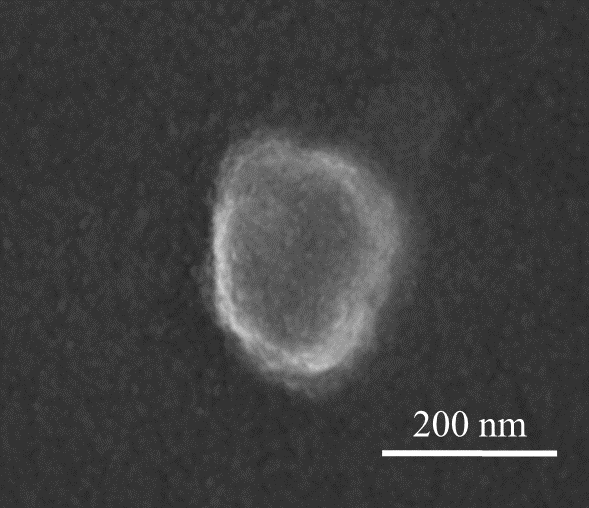


**Fig. S3** High resolution SEM image of Pt particle surrendered by PEDOT:PSS layer.


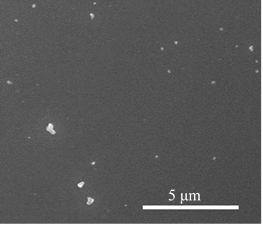


**Fig. S4** SEM images of PEDOT:PSS layer fabricated with high volume ration (20%) of H_2_PtCl_6_.


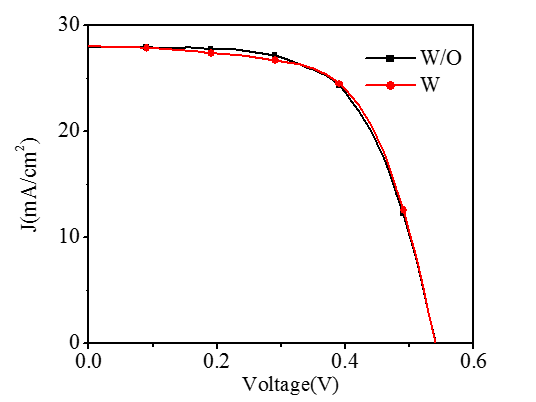


**Fig. S5** J-V characteristics of the hybrid silicon/PEDOT:PSS devices fabricating without and with water addition. The volume ration of water is 10%.
